# Case report: Takotsubo cardiomyopathy and cardiac arrest in a 9-year-old girl with new-onset diabetes presenting with diabetic ketoacidosis: the chicken or the egg?

**DOI:** 10.3389/fendo.2025.1723428

**Published:** 2026-01-07

**Authors:** Sanja Panic Zaric, Vladislav Vukomanovic, Rade Vukovic, Tatjana Milenkovic, Sladjana Todorovic, Katarina Mitrovic, Dimitrije Cvetkovic, Stasa Krasic

**Affiliations:** 1 Endocrinology Department, Mother and Child Health Institute of Serbia, Belgrade, Serbia; 2 Cardiology Department, Mother and Child Health Institute of Serbia, Belgrade, Serbia; 3 Faculty of Medicine, University of Belgrade, Belgrade, Serbia

**Keywords:** cardiac arrest, child, diabetic ketoacidosis, stress cardiomyopahty, Takotsubo cardiomyopathy

## Abstract

**Background:**

Diabetic ketoacidosis (DKA) is an acute and life-threatening complication of diabetes mellitus type 1 (T1DM). There is no published data about the incidence of cardiac arrest in pediatric DKA, but the scarcity of published case reports suggests a very low incidence. Here we present a rare case of a previously healthy 9-year-old girl with new T1DM presenting with severe DKA and influenza infection who developed cardiac arrest, ventricular tachycardia (VT) and stress cardiomyopathy during the initial hours of DKA treatment without any underlying electrolyte disorder, heart disease or hypoglycemia.

**Case report:**

A 9-year-old febrile girl was admitted to our pediatric intensive care unit (PICU) for treatment of severe DKA (pH 6.72, bicarbonate 3.4 mmol/L, glycaemia 28.2 mmol/L, urine ketones 10 mmol/L) with normal electrolyte status. The treatment of severe DKA was promptly started, with the addition of mannitol due to computed tomography (CT) signs of mild initial cerebral swelling. In the seventh hour of DKA treatment, bradycardia developed and, within a minute, progressed to asystolic cardiac arrest with a resultant sudden drop in oxygen saturation and arterial pressure. Immediate measures of cardiopulmonary-cerebral resuscitation were started, and adrenaline and atropine were administered, which resulted in a change from asystole to polymorphic ventricular tachycardia. Two direct current cardioversions were performed, restoring the patient’s sinus rhythm and stabilization. Blood gas analyses showed the persistence of hyperglycemia and severe metabolic acidosis (pH 6.81, HCO3 4.0 mmol/L, glycemia 34.8 mmol/L) without any electrolyte imbalances and further increase in lactate levels. The girl was intubated, and mechanical ventilation was initiated. Echocardiography detected moderately impaired left ventricular systolic function, hypo- and dyskinesia of the interventricular septum. Bicarbonates and inotropic stimulation were administered. The further clinical course was uneventful, with gradual improvement, resolution of ketoacidosis, and restoration of cardiac function. Due to a mild fever and elevated C-reactive protein levels, a PCR test confirmed an infection with the AH3+ influenza virus. She was discharged after 14 days of treatment with insulin and an ACE inhibitor, with normal echocardiography findings.

**Conclusion:**

This case highlights that potentially fatal stress cardiomyopathy and cardiac arrest can unexpectedly occur during the treatment of pediatric severe DKA, even without electrolyte disturbances, brain edema or any history of prior heart disease. Due to these risks, we conclude that all pediatric patients with severe DKA should be treated in the PICU, with continuous ECG monitoring.

## Introduction

Diabetic ketoacidosis (DKA) is an acute and life-threatening complication of diabetes mellitus type 1 (T1DM). Relying on *International Society for Pediatric and Adolescent Diabetes* (ISPAD) guidelines, all three biochemical criteria are required to diagnose DKA: hyperglycemia (blood glucose >11 mmol/L), venous pH < 7.3 or serum bicarbonate <18 mmol/L and ketonemia or ketonuria (1). Due to severe potential complications of DKA, even today, DKA is a leading cause of mortality in children and young people with diabetes. Complications may arise due to the pathologic processes in DKA itself or its management, with the most frequent complications being cerebral edema, hypoglycemia and electrolyte abnormalities (2). High blood sugar, acidosis, systemic stress, catecholamine release, high levels of circulating fatty acids, water deficit and electrolytic alterations can directly harm the heart by causing toxicity, disrupting blood flow to the heart muscle, affecting autonomic functions, and altering the way electrical signals are transmitted through the heart (3). This increases the likelihood of arrhythmias, myocardial stunning and ischemia, which is why monitoring patients with electrocardiograms during an episode of DKA is crucial, as it helps identify arrhythmias and assists in correcting metabolic imbalances (4). Yet, although electrolyte disturbances are frequent in DKA, arrhythmias are scarce (5). There is no published data about the incidence of cardiac arrest in pediatric DKA, but the scarcity of published case reports suggests a very low incidence. Even more so, cardiac arrest in young individuals without underlying heart disease and acute electrolyte disturbances, such as hypokalemia or severe hypoglycemia, is an exceptionally rare complication of DKA (6, 7). Myocardial injury is a severe complication related to increased mortality in patients with severe DKA, accounting for 28% of deaths in adult patients with DKA (8). One possible trigger for the new onset of T1DM presenting with DKA could be human influenza A infection (9). It is recognised that influenza infection is linked to significant cardiovascular complications (10).

Here we present a rare case of a previously healthy 9-year-old girl with newly diagnosed type 1 diabetes presenting with severe DKA and influenza infection who developed cardiac arrest, ventricular tachycardia (VT) and stress cardiomyopathy during the initial hours of DKA treatment without any underlying electrolyte disorder, heart disease, or hypoglycemia. Thus, this case report emphasises the importance of cardiac evaluation and monitoring in a pediatric intensive care unit (PICU) setting in managing severe DKA, even without known heart disease.

## Case presentation

A 9-year-old girl was admitted to our pediatric intensive care unit (PICU) for treatment of severe DKA. She has had polyuria and polydipsia during the past 3 months, with a weight loss of 7kg. Two days before admission, she had a sore throat and was mildly febrile. The night before admission to our centre, her parents noticed deep, laboured breathing and, early in the morning, took her to the emergency room, where she was treated with 40 mg of methylprednisolone and beta-agonist inhalation for suspected bronchial obstruction. She was then referred to the regional hospital, where she was noted to be febrile (38.2°C), mildly dehydrated and sleepy. After a physical exam, she was treated with an intravenous infusion of 750 ml D5W solution. When the blood test results were returned, they indicated severe DKA: glycaemia 33.9 mmol/L, pH 6.8, and she received an additional 300 ml of normal saline i.v. and 15 ml of bicarbonates orally (at the discretion of the local physician).

She didn’t have any prior illness or other health conditions and wasn’t using any medications. There was no recorded family history of endocrine or cardiac conditions. She had a Thai mother and a Serb father.

At the time of admission to our center, she was noted to be dehydrated with Kussmaul’s breathing pattern (RR 30/min), depressed state of consciousness (Glasgow coma score 10/15), tachycardic (HR 150/min) and mildly hypertensive (BP 122/99 mmHg). Venous blood gas analysis and lab results confirmed severe DKA (pH 6.72, bicarbonate 3.4 mmol/L, glycaemia 28.2 mmol/L, urine ketones 10 mmol/L), and levels of electrolytes were within the normal range: K 4.8 mmol/L, Na 133 mmol/L, Mg 0.90 mmol/L, P 1.63 mmol/L (**Table 1**). CRP was mildly elevated (17.7 mg/L), and ECG was normal, with a normal corrected QT interval (QTc) of 0.41 seconds, and chest X-ray was also normal. Continuous noninvasive monitoring was initiated upon admission. Treatment of severe DKA was promptly commenced in accordance with the hospital’s protocol, which aligns with the most recent ISPAD guidelines. Due to a mild fever, blood and urine cultures were performed, and ceftriaxone was initiated. A computed tomography (CT) scan revealed mild initial cerebral swelling in DKA, so mannitol was commenced at the usual dose of 1g/kg body weight.

**Table 1:** Gradual biochemical changes during the DKA treatment.

Values	Admission	7^th^ h (CPR)	9^th^ h	11^th^ h	13^th^ h	15^th^ h	17^th^ h	23^rd^ h
pH	6.72	6.81	7.01	7.07	7.20	7.26	7.29	7.35
Glycaemia (mmol/L)	31.4	34.8	25.8	20.2	16.9	15.3	16.3	18.4
K^+^ (mmol/L)	4.5	4.8	4.6	4.0	3.6	3.3	3.5	3.4
Na^+^ (mmol/L)	137.5	139.6	146.0	147.5	147.3	143.4	145.8	142.5
Lactate (mmol/L)	2.5	1.7	1.1	1.1	1.2	1.1	1.3	1.2

Na, sodium, K, potassium.

**Figure 1:**
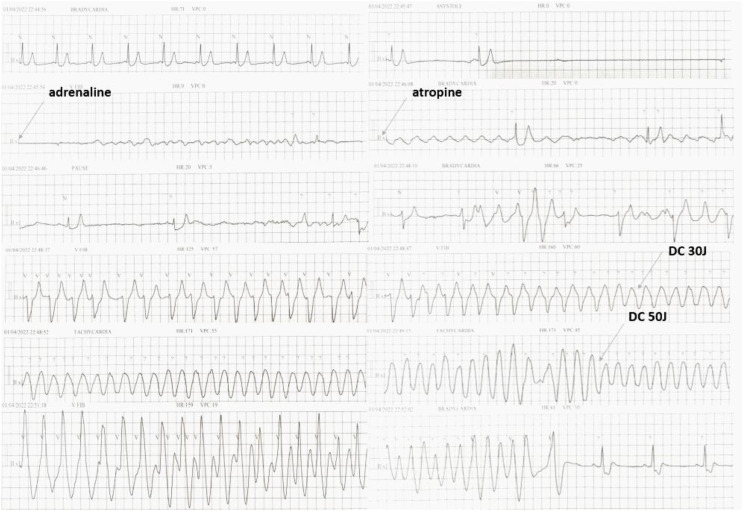
Cardiac arrest in the 7^th^ hour of severe DKA treatment, changing from asystole to monomorphic and polymorphic ventricular tachycardia (torsades des pointes - "twist" around the baseline), ultimately restored to sinus rhythm by DC cardioversion.

During the first 7 hours of in-hospital stay, the patient was hemodynamically stable, with regular capillary refill and no change in mental status. On ECG telemetry, no ectopy and QTc changes were observed. In the seventh hour following the PICU admission and DKA treatment, bradycardia developed and, within a minute, progressed to asystolic cardiac arrest with a resultant sudden drop in oxygen saturation to 60%. Immediate measures of cardiopulmonary-cerebral resuscitation (CPR) were started, and adrenaline and atropine were administered, which resulted in a change from asystole to polymorphic ventricular tachycardia (VT)-*torsades des pointes* (TdP) (**Figure 1**). Two direct current (DC) cardioversions were performed (30J and 50J), restoring and stabilizing the patient’s sinus rhythm. Total CPR time was 7 minutes (**Figure 1**). Blood gas analyses at the moment of resuscitation showed the persistence of hyperglycemia and severe metabolic acidosis (pH 6.81, HCO3 4.0 mmol/L, glycemia 34.8 mmol/L) without any electrolyte imbalances (K 4.8 mmol/L, Na 139.6 mmol/L) and without further increase in the levels of lactate (**Table 1**). The biochemical venous blood lab tests confirmed these results.

During CPR, the girl was quickly intubated, and mechanical ventilation was initiated, maintaining good saturation and mild hypocapnia along with head elevation. Due to neurological deterioration with a seizure, a dose of phenobarbital and an infusion of 3% hypertonic saline were prescribed in addition to mannitol, followed by continuous sedation. An urgent follow-up CT scan was immediately performed, revealing the same findings as on admission, without any signs of brainstem herniation or further progression of brain edema.

**Figure 2:**
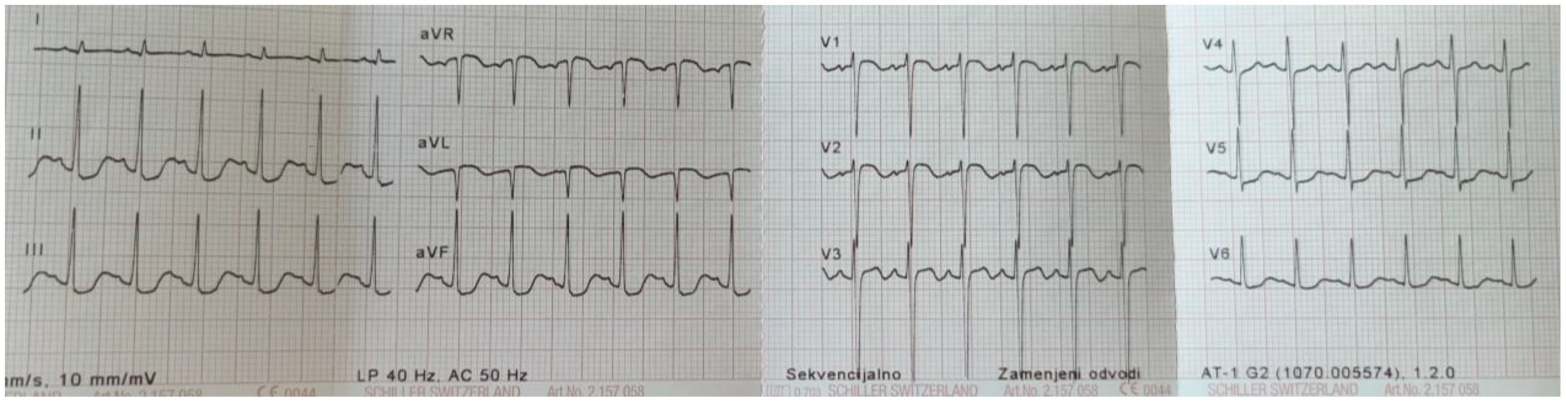
Post-resuscitation ECG revealed ST depression in the inferior and lateral leads, which can indicate post-resuscitation ischemia.

**Figure 3:**
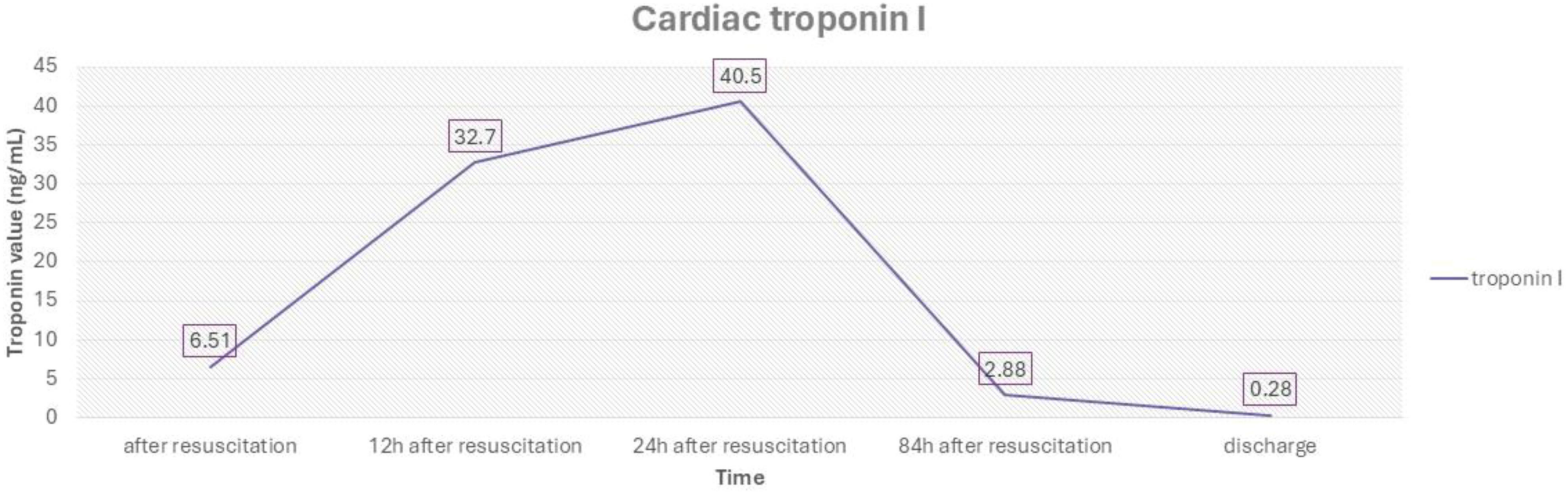
Troponin trends during in-hospital stay.

Due to hypotension following resuscitation, i.v. boluses of normal saline and continuous dopamine infusion (10 mcg/kg/min) were administered. Chest X-ray indicated pulmonary edema, which was most likely cardiogenic. Echocardiography detected left ventricular (LV) systolic dysfunction with an ejection fraction of 44-49%, along with hypokinesia of the interventricular wall and apex with hypercontractility of the basal segments. The heart, coronary vessel structures and other cardiac functions were normal. On the ECG, ST-segment depression in the inferior leads and discrete ST-segment elevation in the right precordial leads were noted (**Figure 2**). The level of brain natriuretic peptide (BNP) was 4705 pg/ml, and the troponin level was 6.51 ng/mL (reference range < 0.1 ng/mL) (**Figure 3**). Due to compromised cardiac contractility, in accordance with the ISPAD guidelines, bicarbonates were administered at the rate of 2 mmol/kg over 2 hours, and inotropic stimulation with continuous dobutamine was added (6 mcg/kg/min). The further clinical course was uneventful, with gradual improvement, resolution of ketoacidosis and extubation within 24 hours. Ultrasound of the chest and abdomen revealed polyserositis. Due to a persistent mild fever and elevated C-reactive protein and procalcitonin levels, antimicrobial therapy was adjusted. Blood and urine cultures remained sterile, and a PCR test confirmed an infection with the AH3+ influenza virus. Screening for T1DM-associated autoimmune diseases was negative.

**Figure 4:**
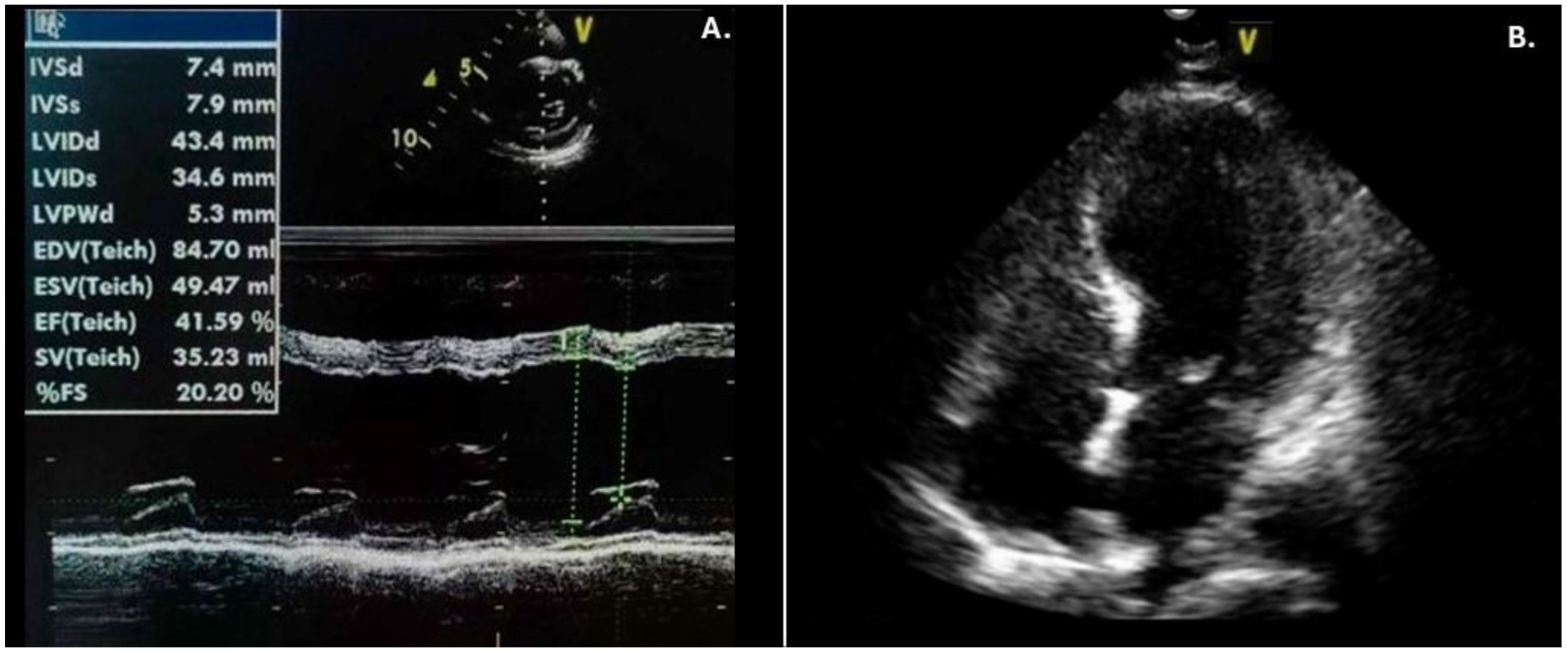
Echocardiography of our patient after resuscitation revealed left ventricular systolic impairment **(A)** with apical dyskinesia **(B)**.

**Figure 5:**
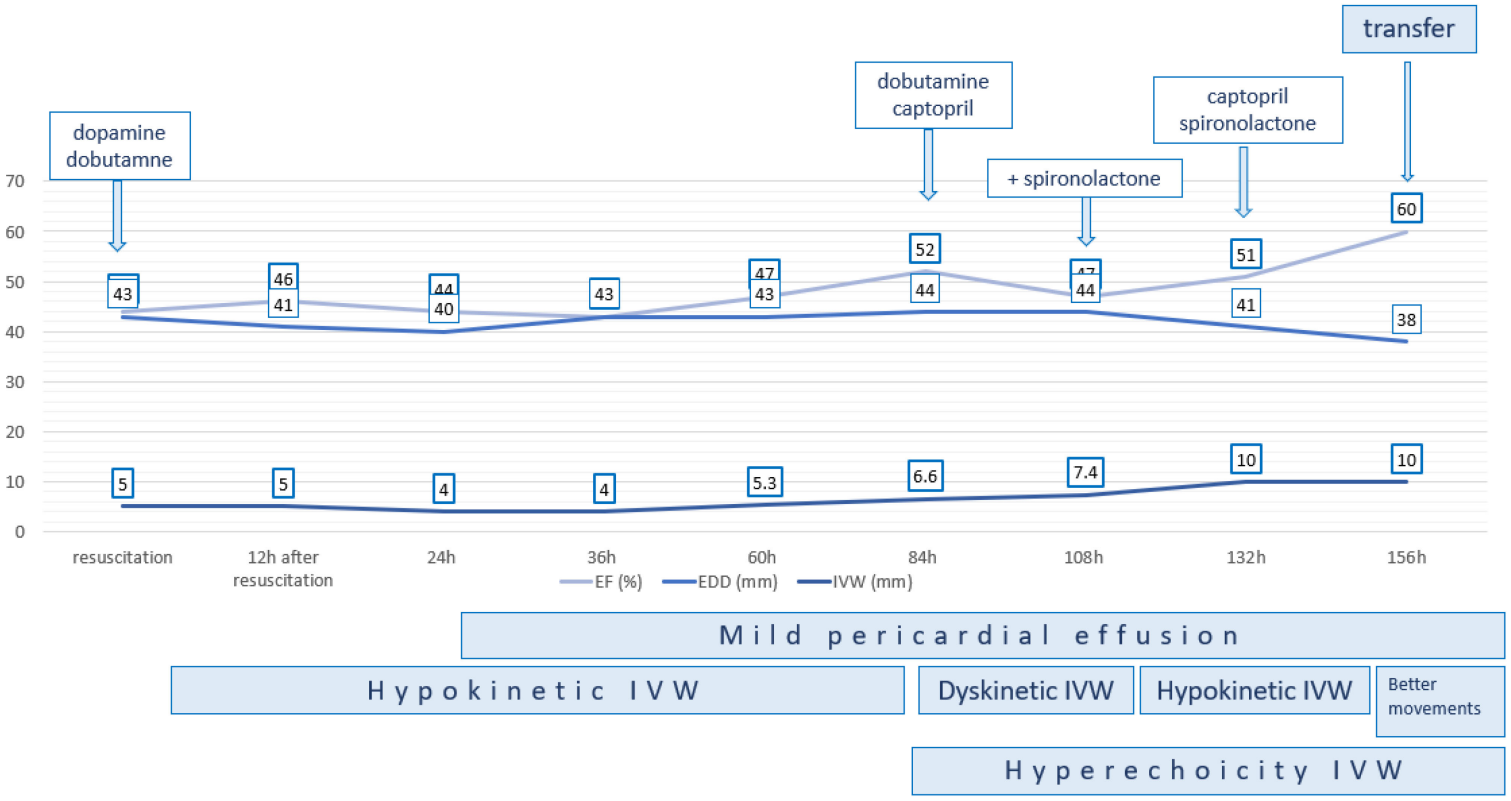
Gradual recovery of the systolic function of the left ventricle. EF, ejection fraction; EDD, end-diastolic diameter; IVW, interventricular wall.

Throughout her stay in the PICU, the patient had dyskinesia and hyperechoic interventricular septal thickening, with gradual recovery of LV systolic function (**Figures 4** and **5**). Dopamine was stopped after 5 days, and dobutamine was stopped one day later. Drugs for chronic heart failure treatment were initiated. After 7 days in the PICU, she was transferred to the cardiology ward and started on a subcutaneous multiple-dose injection insulin regimen.

She was discharged after 14 days of treatment with insulin and an ACE inhibitor, with normal echocardiography findings. After three years of follow-up, the last echocardiography shows normal systolic and diastolic LV function.

## Discussion

We presented a 9-year-old girl with new-onset diabetes and AH3+ influenza infection, presenting with diabetic ketoacidosis, cardiac arrest and stress cardiomyopathy. Yet, at our centre, we have approximately 50–60 admissions of patients with new onset T1DM per year, with up to 38% of these children being newly diagnosed in DKA (11). Almost one-third of these patients (31%) have severe DKA, but this is the first case complicated by cardiac arrest. In individuals with DM in adulthood, acute cardiac decompensation frequently occurs as a result of early-onset atherosclerosis. This complication is much less frequent and, consequently, is given less attention in pediatric cases of DM. Nevertheless, it is essential not to disregard the association between DKA and myocardial dysfunction in children (8). In this case, the definite aetiology of cardiac arrest remains puzzling, but might be a consequence or reason for the “broken heart syndrome”.

Only a few other published cases describe cardiac arrest in the course of pediatric DKA treatment (**Table 2**). All cases had severe acidosis in combination with significant electrolyte imbalance, such as hypophosphatemia, hyperosmolarity, and hypokalemia. Severe electrolyte imbalances and acidosis are the primary causes of the described ECG changes, QTc prolongation, and arrhythmias (5, 18–22). However, arrhythmias are a well-known but rare complication of DKA (23). The accessible data in the pediatric population presents reports of supraventricular tachycardia, Brugada phenocopy, and VT, all without underlying heart disease. Our previously healthy girl experienced asystole and cardiac arrest, followed by TdP during severe DKA treatment. Considering that the admission echocardiography had not been performed, we were unsure whether TdP was a reperfusion arrhythmia or, in combination with asystole, a contributing factor to Takotsubo cardiomyopathy (TCM). However, all electrolytes were within the safe range at the time of admission and immediately before and after resuscitation. The QTc interval was normal on admission and remained so after resuscitation. Additionally, all cases with cardiac arrest experienced DKA signs and symptoms of cerebral oedema, while one of them had severe brain swelling and bilateral uncal herniation. Unfortunately, this patient had four cardiac arrests with asystole and subsequent VT before his diagnosis of brain herniation and PICU admission. This patient withdrew treatment, and the authors highlighted that causes of death related to DKA before receiving medical attention in young patients have encompassed hypovolemic shock, severe acidemia, hyperosmolality, presumed fatal arrhythmias stemming from electrolyte depletion and infectious complications (17).It is well established that various mechanisms in DKA contribute to myocardial injury and poor myocardial performance. Firstly, acidosis can cause cardiac contractile dysfunction (24). Newer research indicates that severe acidemia independently influences myocardial stunning by activating intracellular calcium and contractile proteins (24). In addition to acidosis, acute cardiac decompensation can be associated with severe insulin deficiency and elevation in counter-regulatory hormones (adrenaline, cortisol, and glucagon), which increase the oxygen demand of the myocardium due to altered cardiac metabolism during DKA. Additionally, DKA is a hyperosmolar, hypovolemic state, which causes blood flow disturbances promoting cardiovascular injury (8). This supply/demand mismatch leads to myonecrosis, elevated troponin I levels and can induce “broken heart syndrome” (24). According to literature data, there have been only a few pediatric cases of DKA-induced myocardial dysfunction and cardiomyopathy (**Table 2**), while one 15-year-old boy had coronary artery spasm without functional repercussion (2, 8, 12–15, 25, 26). Takotsubo cardiomyopathy (TCM), also known as stress-induced cardiomyopathy, is defined as an acute, transient LV dysfunction with no evidence of obstructive coronary artery disease on imaging, and might be a consequence of catecholamine-induced coronary spasm (12, 14, 25–27). After cardiac arrest and TdP, the observed ECG changes in our case were a consequence of coronary hypoperfusion. Heightened activity in the sympathetic nervous system in DKA, along with a dense concentration of beta-adrenoceptors in the apex of the heart, induced the classic reduced movement of the apex, which is pathognomonic of stress cardiomyopathy—LV apex ballooning and hypercontractility of the basal segments (25–27). Serial echocardiography examination of our patient after CPR revealed hypo- and dyskinetic movement abnormality of IVW with moderately impaired LV systolic function, and possible TCM with complete resolution in 2 weeks. Despite resolution of ventricular function usually seen within days to weeks, it has been associated with serious complications such as cardiogenic shock, severe cardiac arrhythmias and death (12, 27). Cardiac arrest was observed in up to 6% of patients with TCM, and it was associated with a six-fold increase in short- and long-term mortality (7). Only one adult patient was described with cardiac arrest and TCM during DKA (27), while one previously healthy 12-year-old girl with severe dilated cardiomyopathy during DKA withdrew treatment (16). Another theory suggests that lipotoxicity effects, such as the formation of free fatty acids and micelles in the myocardial plasma membrane, destabilise and rupture the cardiomyocyte membrane (28). This process is exacerbated by high levels of counterregulatory hormones, such as catecholamines, which support the TCM theory.

**Table 2:** Literature review of cardiovascular manifestations in patients with diabetic ketoacidosis.

Paper	Age	Admission	Cardiac decompensation	Symptoms/Signs	Cardiac examination	Follow-up
Pediatric patients with myocardial dysfunction during DKA						
Japitana et al.2013 (12)	13-years; boy	pH 6.778	2^nd^ day	chest pain	ECG: ST-segment elevations w Troponin T 1.37 ng/m MRI - diffuse global hypokinesis, LGE	discharged
Roberts et al., 2009 (13)	9-years; girl	pH 6.86	6 hours	pulmonary oedema	Troponin 0.12 ng/ml ECG: 1) prolonged QTc interval (489 ms); 2) ST depression in the inferior and lateral leads and non-specific T wave abnormality ECHO: global right and left ventricular dysfunction with hypokinetic systolic contraction of the base and mid-section of the left ventricle	discharged
Halloum et Neyadi. 2019 (2)	5-years; girl	pH 6.92	2^nd^ day	respiratory distress hypotension	Troponin – 0.09 µg/L BNP 18,717 ng/L ECG: sinus tachycardia ECHO: EF 33.5%	discharged from the PICU to the pediatric ward.
Strah et at 2021 (14)	15-years; boy	pH 6.94	2^nd^ day	chest pain	ECG: diffuse ST elevation hsTroponin 504.2,523 ng/l ECHO: normal anatomy, biventricular size and systolic function with no focal wall motion abnormalities and no pericardial effusion Coronarography: angiographically normal coronary arteries MRI: no oedema, fibrosis or ischaemia	discharge
Shim et al. 2021 (8)	12-years; girl	pH 7.117	6 hours	epigastric discomfort and mild abdominal pain	Troponin-I 172.0 pg/mL ECG: QTc prologation ECHO: LV EF 29%	discharged
Siddhi et al., 2025 (15)	12-years; girl	pH 7.06	2^nd^ day	poor consciousness, dropping GCS, and hemodynamic compromise	Troponin 758 ng/L BNP 34297 pg/mL ECG: sinus rhythm ECHO: biventricular dysfunction, EF 10–15%	Withdrawn treatment
Lane et al., 2015 (16)	18-years; boy	pH 7.13	admission	lethargy, vomiting, tachypnoea and increasing drowsiness	ECG: elevation in leads II, III and aVF with right bundle branch block hsTrp: 61.1856 ng/L ECHO: global LV hypokinesis with apical akinesis/dyskinesis *pneumomediastinum	discharged
Pediatric patients with cardiac arrest during DKA						
Choi et al 2017 (6)	14-year; girl	pH 6.92 K^+^ 2.6 mmol/L P^2+^ 0.4 mg/dL	4 hours	drowsiness generalised tonic seizure	bradycardia and cardiopulmonary arrest inotropic and ventilator supports	discharged
Grout et al., 2022 (7)	13-year boy	pH 6.99. 7.1 K+ 2.7. 1.9 mmol/L	10 hours	urinary incontinence less responsive (GCS score of 9)	ECG: prolonged corrected QT (616 per machine, 524 per hand calculation) Stable VT; cardiac arrest, intubation and 12 minutes of cardiopulmonary resuscitation	discharged
Fiordalisi et al, 2002 (17)	17-year; boy		Before admission	Cardiac arrest + CPR		Withdrawn treatment

GSC, Glasgow coma score; CPR, cardio-pulmonary resuscitation, LV, left ventricle, PICU, pediatric intensive care unit.

Additionally, a state of systemic inflammation and infection can increase free radical production, thereby inhibiting contractile proteins and causing myocardial stunning (28). In the presented case, influenza infection likely contributed to the new-onset DKA and T1DM and may have contributed to stress cardiomyopathy in our patient. The burden of influenza infection is predominantly determined by the recognition of widely acknowledged respiratory manifestations. Thus, it’s essential not to overlook extrapulmonary complications. The connection between cardiac conditions and influenza infection is intricate. While human influenza typically affects the respiratory tract, complications such as myocarditis and pericarditis are widely acknowledged. Also, influenza infection may trigger arrhythmias, cardiac ischemia, infarction, cardiomyopathy and cardiac arrest (10, 29). While the clinical course was consistent with stress cardiomyopathy, fulminant myocarditis cannot be entirely excluded in the absence of cardiac MRI or histopathologic confirmation. According to the European Society of Cardiology (ESC), two diagnostic criteria must be fulfilled in asymptomatic patients to diagnose clinically suspected myocarditis (30). ECG finding of our patient on admission was normal, but she developed VT after the administration of adrenaline and atropine due to asystole. The troponin level was elevated after reanimation; it may be a consequence of the resuscitation or myocarditis. Additionally, echocardiographic findings after resuscitation indicate impaired LV systolic function, interventricular wall and apex hypokinesia, and hypercontractility of the basal segments, with normal LV dimensions, a characteristic finding after resuscitation or, eventually, of acute, rapidly progressive fulminant myocarditis. The prompt decrease and normalisation of troponin levels, along with the improvement in LV systolic function, even in the absence of immunomodulatory therapy, do not support the diagnosis of acute fulminant myocarditis. However, since the diagnosis of TCM was considered, myocardial biopsy and cardiac magnetic resonance were not performed. Additionally, lactate levels remained within normal limits throughout the arrest and immediate post-resuscitation periods, suggesting early recognition, short CPR duration, and preserved perfusion before arrest (in the absence of signs of heart failure). This finding also speaks strongly against fulminant myocarditis.

However, the very fact that a 9-year-old girl without any known underlying heart condition or other warning signs developed asystolic cardiac arrest followed by TdP during the first hours of DKA treatment emphasises the need for baseline cardiology examination in all DKA patients with severe acidosis (not just for quick potassium level assessment), cardiac monitoring and treatment of all patients with severe DKA in the ICU setting. Continuous cardiovascular monitoring in the ICU can provide early recognition of rhythm disturbances, rapid response capability, detection of signs of cerebral oedema, and differentiation between respiratory and cardiac causes of sudden deterioration.

## Conclusion

We presented the case of a child with cardiac arrest and TCM in the setting of severe new-onset DKA, without significant electrolyte disturbances or previous heart disease, most likely triggered by the influenza infection. This case highlights that potentially fatal cardiac arrest and TCM can unexpectedly occur during the treatment of pediatric DKA, even without electrolyte disturbances, brain edema or any history of prior heart disease. All pediatric patients with severe DKA should be treated in the PICU, with continuous ECG monitoring due to these risks. Additionally, this case highlights the importance of routine ECG and echocardiography upon admission in children with severe DKA, not only for the prompt assessment of potassium disturbances but also to identify any abnormalities that may predispose to serious arrhythmias. Additionally, if cardiac arrhythmia occurs during DKA treatment, having insight into the pre-treatment admission and ECG would be crucial for informed decision-making. It is essential to raise awareness about the early signs and symptoms of T1DM and the significance of DKA as a severe, life-threatening complication.

## Data Availability

The raw data supporting the conclusions of this article will be made available by the authors, without undue reservation.

## References

[1] Glaser N Fritsch M Priyambada L Rewers A Cherubini V Estrada S . ISPAD clinical practice consensus guidelines 2022: Diabetic ketoacidosis and hyperglycemic hyperosmolar state. Pediatr Diabetes. (2022) 23:835–56. doi: 10.1111/pedi.13406, PMID: 36250645

[2] Halloum A Al Neyadi S . Myocardial dysfunction associated with diabetic ketoacidosis in a 5-year-old girl. SAGE Open Med Case Rep. (2019) 7:2050313X19847797. doi: 10.1177/2050313X19847797, PMID: 31105952 PMC6503591

[3] Carrizales-Sepúlveda EF Ordaz-Farías A Vera-Pineda R Rodríguez-Gutierrez R Flores-Ramírez R . Comprehensive echocardiographic and biomarker assessment of patients with diabetic ketoacidosis. Cardiovasc Diabetol. (2024) 23:385. doi: 10.1186/s12933-024-02471-0, PMID: 39468588 PMC11520802

[4] Carrizales-Sepúlveda EF Vera-Pineda R Jiménez-Castillo RA Violante-Cumpa JR Flores-Ramírez R Ordaz-Farías A . The heart in diabetic ketoacidosis: A narrative review focusing on the acute cardiac effects and electrocardiographic abnormalities. Am J Med Sci. (2021) 361:690–701. doi: 10.1016/j.amjms.2020.11.030, PMID: 33941367

[5] Faruqi TA Hanhan UA Orlowski JP Laun KS Williams AL Fiallos MR . Supraventricular tachycardia with underlying atrial flutter in a diabetic ketoacidosis patient. Clin Diabetes. (2015) 33:146–9. doi: 10.2337/diaclin.33.3.146, PMID: 26203208 PMC4503945

[6] Choi HS Kwon A Chae HW Suh J Kim DH Kim HS . Respiratory failure in a diabetic ketoacidosis patient with severe hypophosphatemia. Ann Pediatr Endocrinol Metab. (2018) 23:103–6. doi: 10.6065/apem.2018.23.2.103, PMID: 29969883 PMC6057019

[7] Grout S Maue D Berrens Z Swinger N Malin S . Diabetic ketoacidosis with refractory hypokalemia leading to cardiac arrest. Cureus. (2022) 14:e23439. doi: 10.7759/cureus.23439, PMID: 35494963 PMC9038207

[8] Shim HJ Yoo BM Jin SM Kang MJ . Myocardial injury in a pediatric patient with diabetic ketoacidosis: A case report. Med (Baltimore). (2021) 100:e25702. doi: 10.1097/MD.0000000000025702, PMID: 33907151 PMC8084016

[9] Nishioka Y Noda T Okada S Myojin T Kubo S Higashino T . Association between influenza and the incidence rate of new-onset type 1 diabetes in Japan. J Diabetes Investig. (2021) 12:1797–804. doi: 10.1111/jdi.13540, PMID: 33660948 PMC8504904

[10] Filgueiras-Rama D Vasilijevic J Jalife J Noujaim SF Alfonso JM Nicolas-Avila JA . Human influenza A virus causes myocardial and cardiac-specific conduction system infections associated with early inflammation and premature death. Cardiovasc Res. (2021) 117:876–89. doi: 10.1093/cvr/cvaa117, PMID: 32346730 PMC7898948

[11] Vukovic R Jesic MD Vorgucin I Stankovic S Folic N Milenkovic T . First report on the nationwide incidence of type 1 diabetes and ketoacidosis at onset in children in Serbia: a multicenter study. Eur J Pediatr. (2018) 177:1155–62. doi: 10.1007/s00431-018-3172-4, PMID: 29774417

[12] Japitana MG Abdeljaber AH Basnet S . Stress cardiomyopathy in pediatric diabetic ketoacidosis. Cardiovasc Endocrinol. (2012) 2:31–4. doi: 10.1097/XCE.0b013e328360b104

[13] Roberts KD Levin DL . Diabetic ketoacidosis, respiratory distress and myocardial dysfunction. BMJ Case Rep. (2009) 01:1530. doi: 10.1136/bcr.01.2009.1530, PMID: 22132021 PMC3028211

[14] Strah D Seckeler M Mendelson J . Coronary artery spasm in a 15-year-old male in diabetic ketoacidosis. Cardiol Young. (2021) 31:1507–9. doi: 10.1017/S1047951121000780, PMID: 33719987

[15] Siddhi G Manojkumar P Shradha SR Balkrishna G Sampada TA Neha T . Dilated cardiomyopathy in adolescents with diabetic ketoacidosis: A case report. J Pediatr Crit Care. (2025) 12:70–4. doi: 10.4103/jpcc.jpcc_77_24

[16] Lane AS Champion B Orde S Dravec D . Diabetic ketoacidosis due to fulminant type 1 diabetes: A rare subtype of type 1 diabetes leading to unusual sequelae. J Intensive Care Soc. (2015) 16:64–70. doi: 10.1177/1751143714551249, PMID: 28979377 PMC5593281

[17] Fiordalisi I Harris GD Gilliland MG . Prehospital cardiac arrest in diabetic ketoacidemia: why brain swelling may lead to death before treatment. J Diabetes Complications. (2002) 16:214–9. doi: 10.1016/s1056-8727(01)00177-5, PMID: 12015191

[18] Youssef OI Farid SM . QTc and QTd in children with type 1 diabetes mellitus during diabetic ketoacidosis. ISRN Pediatr. (2012) 2012:619107. doi: 10.5402/2012/619107, PMID: 23209932 PMC3503306

[19] Alanzalon RE Burris JR Vinocur JM . Brugada phenocopy associated with diabetic ketoacidosis in two pediatric patients. J Electrocardiol. (2018) 51:323–6. doi: 10.1016/j.jelectrocard.2017.10.017, PMID: 29174707

[20] Finn BP Fraser B O’Connell SM . Supraventricular tachycardia as a complication of severe diabetic ketoacidosis in an adolescent with new-onset type 1 diabetes. BMJ Case Rep. (2018) 2018:bcr2017222861. doi: 10.1136/bcr-2017-222861, PMID: 29545427 PMC5878340

[21] Shati AA Al-Asmari OJ Alhayani AA Alqahtani YA Alshehri SA Alhelali IA . Supraventricular tachycardia associated with severe diabetic ketoacidosis in a child with new-onset type 1 diabetes mellitus. Cardiol Young. (2022) 32:1677–80. doi: 10.1017/S1047951122000208, PMID: 35094738

[22] McGreevy M Beerman L Arora G . Ventricular tachycardia in a child with diabetic ketoacidosis without heart disease. Cardiol Young. (2016) 26:206–8. doi: 10.1017/S1047951115000621, PMID: 26446852

[23] Abbas Q Arbab S Haque AU Humayun KN . Spectrum of complications of severe DKA in children in pediatric Intensive Care Unit. Pak J Med Sci. (2018) 34:106–9. doi: 10.12669/pjms.341.13875, PMID: 29643888 PMC5856992

[24] Kaefer K Botta I Mugisha A Berdaoui B De Bels D Attou R . Acute coronary syndrome and diabetic keto acidosis: the chicken or the egg? Ann Transl Med. (2019) 7:397. doi: 10.21037/atm.2019.07.38, PMID: 31555711 PMC6736820

[25] Escaño L Desai P Chaudhry S . From hyperglycemia to broken heart syndrome: A case of diabetic ketoacidosis-induced takotsubo cardiomyopathy. Cureus. (2024) 16:e64907. doi: 10.7759/cureus.64907, PMID: 39156256 PMC11330627

[26] Abbas A Patel N Kazmi R Mirza N Miller R Correia J . Diabetic ketoacidosis-induced cardiomyopathy and reversible dialysis-dependent renal failure with successful outcome: A report of a rare case. Cureus. (2022) 14:e31711. doi: 10.7759/cureus.31711, PMID: 36569685 PMC9770522

[27] Teferi AM Paz H Bankowski S Rahimi M Zaremski L . Takotsubo cardiomyopathy in a young patient presenting as cardiac arrest and cardiogenic shock. Cureus. (2024) 16:e61560. doi: 10.7759/cureus.61560, PMID: 38962651 PMC11221618

[28] Moller N Foss AC Gravholt CH Mortensen UM Poulsen SH Mogensen CE . Myocardial injury with biomarker elevation in diabetic ketoacidosis. J Diabetes Complications. (2005) 19:361–3. doi: 10.1016/j.jdiacomp.2005.04.003, PMID: 16260354

[29] Jeyanathan T Overgaard C McGeer A . Cardiac complications of influenza infection in 3 adults. CMAJ. (2013) 185:581–4. doi: 10.1503/cmaj.110807, PMID: 23549966 PMC3626810

[30] Schulz-Menger J Collini V Gröschel J Adler Y Brucato A Christian V . 2025 ESC Guidelines for the management of myocarditis and pericarditis. Eur Heart J. (2025) 46:3952–4041. doi: 10.1093/eurheartj/ehaf192, PMID: 40878297

